# Does Root Tensile Strength Exhibit Seasonal Variation? Evidence from Two Herbaceous Species

**DOI:** 10.3390/plants14192957

**Published:** 2025-09-24

**Authors:** Kang Ji, Chaochao Deng, Luping Ye, Yi Liu, Feng Liu, Zhun Mao, Juan Zuo

**Affiliations:** 1 Wuhan Botanical Garden, Chinese Academy of Sciences, Wuhan 430074, China; jikang17@mails.ucas.ac.cn (K.J.); dengchaochao20@mails.ucas.ac.cn (C.D.); yeluping@wbgcas.cn (L.Y.); liuyi@wbgcas.cn (Y.L.); liufeng@wbgcas.cn (F.L.); 2 University of Chinese Academy of Sciences, Beijing 100049, China; 3 Danjiangkou Wetland Ecosystem Field Scientific Observation and Research Station, Chinese Academy of Sciences & Hubei Province, Wuhan 430074, China; 4 Univ Montpellier, AMAP, INRAE, CIRAD, CNRS, IRD, 34000 Montpellier, France

**Keywords:** root mechanical trait, root tensile strength, seasonal variation, soil water content, slope stability

## Abstract

Root tensile strength (*T_r_*) is a fundamental root mechanical trait and serves as a key parameter for assessing the contribution of vegetation to slope stability. *T_r_* is known to exhibit high intraspecific variability, but whether *T_r_* varies with season remains unclear. Here, we investigated the seasonal variation in *T_r_* in two commonly seen herbaceous species, i.e., *Artemisia argyi* and *Cirsium setosum*, both of which can be future candidates for revegetating species along roadsides in temperate and subtropical regions. We examined the *T_r_* of their first- (closest to the stem base) and third-order lateral roots sampled in the southwest of Henan, China, in two distinct periods: September (late growing season) and December (dormant season). We found that the *T_r_* of the thicker, first-order roots in September was significantly greater than that in December. However, such seasonal variation was not found for the thinner third-order roots. When fitting the relationship between *T_r_* and root diameter using a two-parameter power law equation, the calibrated equation using the data collected in September led to a marked predictive bias to the data collected in December. All the above patterns were consistent for both species. Soil moisture, which exhibited strong seasonal variation in the study area, might be the key cause of variation in *T_r_*. Our study is among the first to demonstrate seasonal variation in root mechanical traits, indicating that season potentially plays a non-negligible role in impacting soil reinforcement and slope stability by modifying roots’ mechanical quality.

## 1. Introduction

Root tensile strength (*T_r_*, in MPa) is the maximum tensile force (*F_r_*, in N) required per root cross-sectional area to cause root failure and represents the intrinsic ability of the root to resist external forcing in tension [1,2]. *T_r_* is one of the most important root mechanical traits and plays a vital role in assessing the contribution of vegetation to slope stability [3,4]. For example, *T_r_* is an essential parameter of models that quantify soil reinforcement due to roots and slope stability [1,5,6]. *T_r_* can sometimes be used directly as an indicator when selecting and evaluating plants for mitigating erosion and shallow landslides [6,7,8].

It is well-known that *T_r_* exhibits strong inter- and intra-variability, and exploring the sources of the variations in *T_r_* has drawn increasing research attention [1,6]. Besides the significant interspecific variation in *T_r_*, as shown in many studies, there is also enormous intraspecific variation in tensile strength, which can be greater than the interspecific variation [1,9]. However, the magnitude of the intraspecific variation and the underlying mechanisms still remain inadequately understood.

Within the same plant species, previous studies have shown that *T_r_* is regulated by multiple biotic and abiotic factors [4,10,11]. Among the biotic factors, root diameter (*d*, in mm) is the best-known one, and therefore, *T_r_* is often characterized as a function of diameter following a two-parameter power law relationship [12,13,14,15,16]. In general, a thicker root with a larger diameter needs a greater tensile force (i.e., *F_r_*) for the root to break. In contrast, *T_r_*, regarded as a standardized *F_r_*, usually shows a decreasing trend with increasing diameter, although there are also cases showing a plateau or increasing trend [9,12,17]. Compared to diameter, increasing studies show that architectural traits, such as root order, which can better reflect root age, functional type, secondary development and chemical composition, may better explain intraspecific variation in *T_r_* [1,9,10,11,18].

Compared to biotic factors, studies on the relationship between *T_r_* and abiotic factors are relatively scarce. In the limited number of studies, *T_r_* is primarily found to be influenced by soil environmental factors, including soil water conditions [10,19,20]. For example, Zhu et al. [20] found that the *T_r_* of young trees of *Symplocos setchuensis* dropped by approximately 15% with increasing soil moisture. Moreover, Zhu et al. [20] evidenced an interaction effect between the effect of soil moisture and that of root diameter, as the *T_r_* and *F_r_* of thicker roots tended to decline more with increasing soil moisture than those of thinner roots. Loades et al. [10] found that the relationship between the *T_r_* and root diameter of Barley (*Hordeum vulgare*) was weakened in waterlogging conditions compared to non-waterlogging conditions. While previous studies have examined the response of *T_r_* to a multitude of biotic and abiotic factors, the measurements are typically carried out within the same time period (e.g., season). To the best of our knowledge, how *T_r_* varies in different seasons has been little studied (but see Rossi et al. [21]). Soil environments can undergo large changes in terms of temperature, humidity and bulk density across seasons [22]. Therefore, it is plausible that root mechanical traits that are subjected to changes in soil environments may also show seasonal variation. As probably one of the first studies investigating seasonal variation in *T_r_*, Rossi et al. [21] reported that *Cynara cardunculus* exhibited a higher *T_r_* in June compared to October. However, *T_r_* during the dormant season, particularly in winter, has not yet been investigated or compared with data from the growing season. Moreover, the root samples analyzed by Rossi et al. [21] were obtained from plants grown under cultivated conditions, while roots from naturally occurring vegetations have not yet been explored.

Herbaceous plants are widely used in revegetation practices over geotechnical infrastructures, such as road embankments, mines and riverbanks due to their strong capacity to provide the ecosystem service of soil and water conservation [7,23]. Although their roots are generally weaker in mechanical quality than those of woody species [13,24], herbaceous plants can colonize soil more quickly with their dense and relatively homogenous roots. At the same time, herbaceous plants bring minimal surcharge to soil, which can be beneficial for slope stability. However, the growth and lifespan of herbaceous plants are strongly influenced by the seasons [25]. The current assessment of their contributions to slope stability often relies on measurements during the growing season, while whether such contributions can be maintained in the dormant season is overlooked. So far, the data of root mechanical traits in the dormant season are extremely scarce, probably due to the difficulty of field sampling. Could this negligence of dormant season data have significant implications for ecological management? This is an important question, as many shallow landslides can occur outside the growing season in temperate regions [26].

In this study, we aim to investigate the seasonal variation in *T_r_* in two commonly seen herbaceous species, i.e., *Artemisia argyi* and *Cirsium setosum*, both of which can be future candidates for revegetating species along roadsides in temperate and subtropical regions. More precisely, by sampling in situ the roots of the species in the southwest of Henan, China, we compare the *T_r_* of their first- (closest to the stem base) and third-order lateral roots in two distinct periods: September (late growing season) and December (dormant season). Our questions (Q) and associated hypotheses (H) are as follows:

 Q1: Does *T_r_* differ between the growing and dormant seasons? We expect that *T_r_* in the dormant season is significantly lower than that in the growing season (H1).

 Q2: Does root size (proxied by root diameter and order) modulate the seasonal effect on *T_r_*? We expect that the seasonal variation is less pronounced in thicker, first-order roots compared to thinner, third-order roots (H2).

 Q3: Does the season alter the relationship between *T_r_* and *d*, as well as the parameters of its power law model fit? We expect that the *T_r_* versus *d* relationship differs significantly between the growing and dormant seasons and, accordingly, that the observed *T_r_* in the dormant season cannot be reliably predicted by the calibrated model based on the growing season data (H3).

## 2. Results

### 2.1. Seasonal Variation in Soil and Root Characteristics

**Figure 1:**
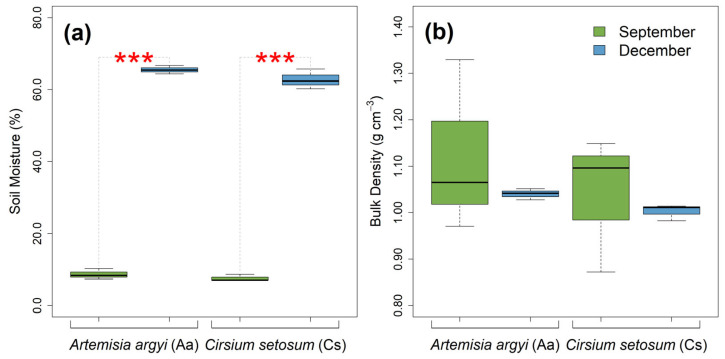
Soil moisture (**a**) and bulk density (**b**) for *Artemisia argyi* and *Cirsium setosum* in September (late growing season) and December (dormant season). Significance code: *** = *p* < 0.001.

Soil moisture in September was significantly lower than that in December, by 86.78% at the *A. argyi* sites and 87.99% at the *C. setosum* sites (*t*-test, *p* < 0.001) (Figure 1). Soil bulk density showed no significant seasonal variation with a mean value of 1.05 g cm^−3^ across all sites.

**Figure 2:**
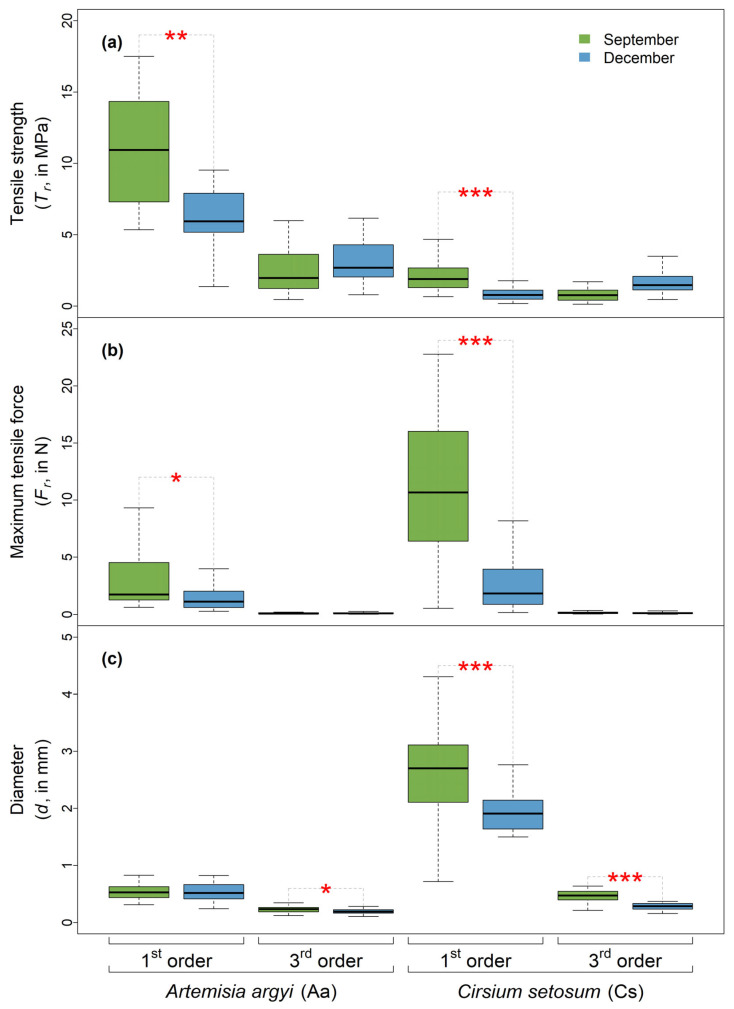
Seasonal variation in root tensile strength (*T_r_*; (**a**)), maximum tensile force (*F_r_*; (**b**)) and diameter (*d*; (**c**)). The terms “1st order” and “3rd order” represent the two root orders, and colors indicate September (late growing season) and December (dormant season), respectively. Significance code: *** = *p* < 0.001; ** = *p* < 0.01; * = *p* < 0.05. Outliers are excluded from the boxplots; see Figure S1 in the Supplementary Materials for the version with outliers.

For both species, the first-order roots were significantly weaker in December compared to September (*t*-test, *p* < 0.01 for *A. argyi* and *p* < 0.001 for *Cirsium setosum*; Figure 2a). For *A. argyi*, the mean *T_r_* of the first-order roots in December was 43.61% weaker than that in September, while for *C. setosum*, such a difference reached 58.70%. However, for third-order roots, no significant seasonal variation in *T_r_* was observed (Figure 2a).

### 2.2. Comparison of Predicted and Observed Root Tensile Strength in Dormant Season

**Figure 3:**
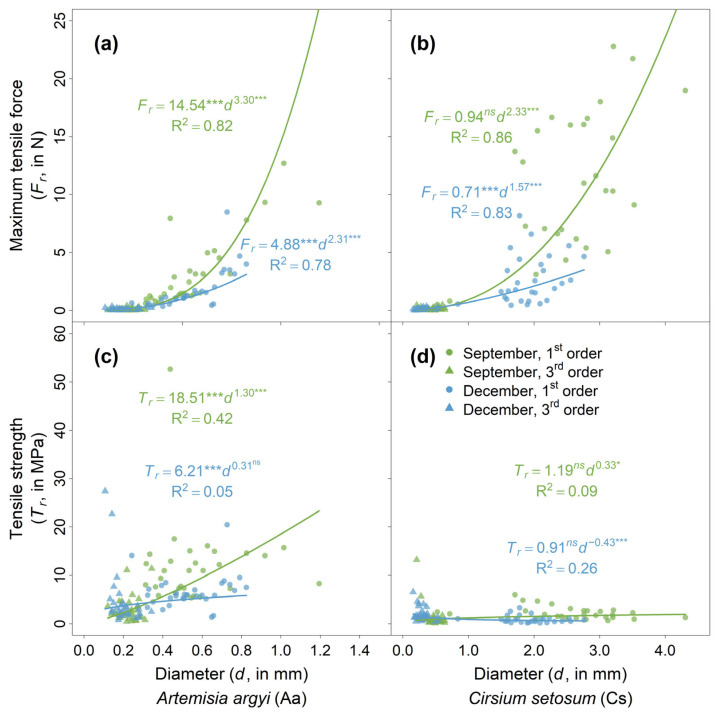
Power law fitting of root diameter with maximum tensile force (*F_r_*; (**a**,**b**)) and tensile strength (*T_r_*; (**c**,**d**)). Symbols of different colors and shapes represent different seasons (warm green = September; light blue = December) and root orders (round = first-order roots; triangle= third-order roots), respectively. Both first- and third-order roots were used to fit equations for each species and season. Significance code for coefficients: *** = *p* < 0.001; * = *p* < 0.05; *ns* = not significantly different from zero.

*F_r_* increased with root diameter in a distinct non-linear pattern, which was well described by a positive power law for both species in September and December (with R^2^ values varying from 0.78 to 0.86; Figure 3a,b). As diameter increased, the *F_r_* of roots in September, as well as the corresponding power law curve, showed a steeper increase compared to that in December, and this was true for both species.

Compared to *F_r_*, the relationships between *T_r_* and diameter showed more complex patterns (Figure 3c,d), including (i) a positive relationship that was well fitted by a power law (i.e., *A. argyi* in September), (ii) a positive relationship that could not be well described by a power law (i.e., *A. argyi* in December) and (iii) no clear relationships with poor power law fit (i.e., *C. setosum* in both seasons).

**Figure 4:**
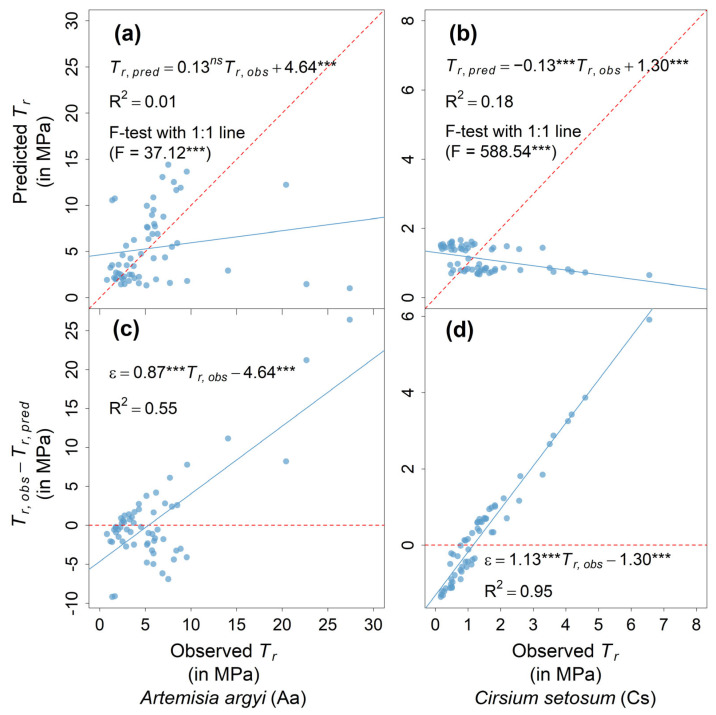
Comparison of predicted and observed root tensile strength (*T_r_*) in December and analysis of model residuals for *Artemisia argyi* (**a**,**c**) and *Cirsium setosum* (**b**,**d**), respectively. Red dotted line represents either 1:1 line (**a**,**b**) or zero residual line (**c**,**d**). In (**a**,**b**), F-value represents result of F-test comparing regression model to 1:1 line. Significance code: *** = *p* < 0.001; *ns* = not significantly different from zero.

Consequently, the power law equation calibrated using the September data performed poorly in predicting *T_r_* in December for both species (Figure 4). This was represented by the pronounced deviation of the regression slope between the predicted and observed *T_r_* from the 1:1 line (Figure 4a,b). Most predicted values exceeded the observed ones, consistently falling above the 1:1 line (Figure 4a,b), with F = 37.12 (*p* < 0.001) for *A. argyi* and F = 588.54 (*p* < 0.001) for *C. setosum*. In addition, the residuals showed a clear linear and positive relationship with the observed *T_r_* (Figure 4c,d), indicating systematic overestimation and substantial deviation from the zero residual line (Figure 4c,d).

## 3. Discussion

As the most extensively studied root mechanical trait, *T_r_* has been found to show strong intraspecific variation, depending on a multitude of factors related to roots (e.g., age and type [1,9]) and the environment (e.g., soil moisture and altitude [20,27]). Here, we found that the thick, first-order roots of the same species in December were significantly weaker (i.e., lower *T_r_*) than those in September (Figure 2a). While such findings validate our H1, they also provide what is likely one of the first pieces of evidence that seasonality is a contributing factor to intraspecific variation in *T_r_*.

Since *T_r_* is directly proportional to *F_r_* and inversely proportional to the root cross-sectional area (Equation (1)), a decrease in *T_r_* may directly result from a reduction in *F_r_*, an increase in root diameter (*d*) or a combination of both. Interestingly, we found that the root diameter in December was either not significantly different from that in September or was significantly smaller (which would theoretically contribute to an increase in *T_r_*; Figure 2c). This suggests that the decrease in *T_r_* observed in December is most likely due to a decline in *F_r_*. Indeed, we observed a greater decline in the *F_r_* of first-order roots in December (Figure 2b). In our case study, the significant environmental factor contributing to the reductions in both *F_r_* and *T_r_* is likely the seasonal change in soil moisture. In December, soil moisture was much higher than that in September due to the lower temperature, the weaker evaporation and the higher river water levels during the sampling time in December (Figure 1a). Previous studies, such as those by Zhu et al. [20], Hales and Miniat [19] and Boldrin et al. [17], have shown that increases in soil and root water content significantly reduce root mechanical strength. Similarly, as one of the first studies investigating seasonal variation in *T_r_*, Rossi et al. [21] also attributed the higher *T_r_* observed in June compared to October to reduced water soil water content during the summer. The major reason for which roots are weaker (i.e., lower *T_r_* and *F_r_*) in wet soils is that water may act as a plasticizer that can reduce internal friction between cells and tissues. Conversely, dry soils result in the partial dehydration of root tissues, leading to an increase in cell wall stiffness.

It should be noted that our study differs from previous studies. Boldrin et al. [17], Hales and Miniat [19], Zhu et al. [20], Hales et al. [28] and Rossi et al. [21] may have measured roots that were not fully saturated and had varying water contents, while we measured fresh and saturated roots with the theoretically comparable water content, since the root–soil composites were immersed in water for at least 24 h. Therefore, the differences lie in the water content of the roots in their in situ growth environments. Here, besides soil abiotic factors, we speculate that the dynamics of soil herbivores, as one of the external environmental conditions surrounding roots during the growing and non-growing seasons, may be a factor influencing these changes. *T_r_* is sensitive to tissue damages or imperfections [9], which can be induced by soil-borne pathogenic microorganisms and soil animals, such as nematodes and root-feeding insects (e.g., beetle larvae). The soil environment in September was extremely dry (only about 15%), likely resulting in smaller populations of soil-dwelling organisms. In contrast, the roots in December were in a much wetter environment, which might cause a different abundance or diversity of soil microbial and fauna communities. We also found that seasonal effects on root mechanical traits were significant for coarse, first-order roots but not for fine, third-order roots. This is in contrast to our second hypothesis, which predicted that finer roots would exhibit higher *T_r_* values and be more sensitive to environmental variation. Two possible explanations may explain this unexpected result:

First, our data do not follow the commonly reported pattern in the literature that *T_r_* increases as root diameter decreases. Instead, our results resemble those of a few previous studies, such as those by Mao et al. [1,9] and Boldrin et al. [29], which observed that smaller roots in certain species tended to have lower *T_r_* (and *F_r_*) values. This trend likely reduces the statistical sensitivity needed to detect seasonal effects in finer roots.

Second, although finer roots are theoretically more responsive to environmental changes within a given period, they also tend to have much shorter lifespans. Compared to larger roots that remain in the soil for longer durations, finer roots may have less exposure time to environmental disturbances such as herbivory by soil-dwelling organisms, which may ultimately reduce the observable seasonal variation in their mechanical traits.

Most existing root mechanical data in the literature are collected during the growing season (see the studies in a meta-analysis in Mao et al. [13] as examples). Our model demonstrates that mechanical equations calibrated based on growing season data fail to accurately predict *T_r_* during the dormant season. This finding validates Hypothesis 3. Given that root mechanical strength is generally higher in the growing season than in the dormant season, using growing season data to represent dormant season conditions is likely to result in an overestimation of the roots’ contribution to soil reinforcement and slope stability. Therefore, future studies on root reinforcement and slope stability should carefully account for seasonal variation as a potentially important factor in ecological engineering models. Moreover, since the *T_r_* measured in the growing season cannot reliably predict *T_r_* in the dormant season, this opens a new avenue for future research on root trait data during the dormant season, which are so far very scanty.

## 4. Materials and Methods

### 4.1. Study Site and Model Species

This study was conducted in the catchment of the Danjiang River. The sampling sites (33°2′–33°4′ N, 111°13′–111°15′ E) were located in Xichuan County, Nanyang, Henan Province, China (Figure 1a), and were approximately 3.5–5 km away from the Danjiangkou Wetland Ecosystem Field Scientific Observation and Research Station, Chinese Academy of Sciences. The region has a subtropical monsoon climate [30]. The mean annual temperature around the sampling site is approximately 15.8 °C, and the average annual precipitation is approximately 804 mm, with most rainfall occurring between June and September [31].

**Figure 5:**
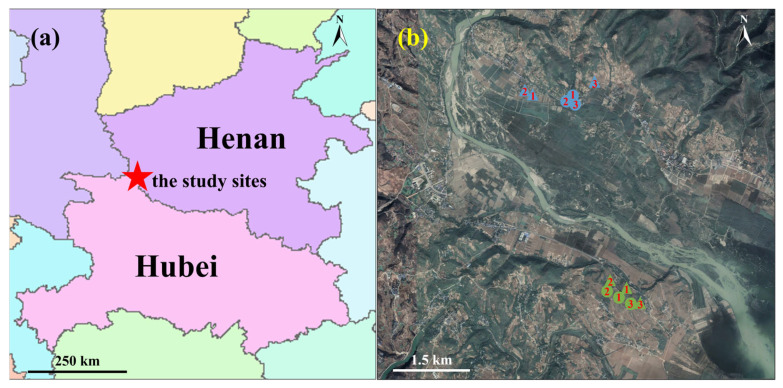
A distribution map of sampling sites: (**a**) the location of the catchment of the study site; (**b**) the distribution of sampling sites. In (**b**), warm green indicates the sampling points in September, while cool blue indicates the sampling points in December; circles represent sampling sites for *Artemisia argyi*, while triangles represent sampling sites for *Cirsium setosum*. The sequence numbers of the sites indicated correspond to those in Table 1. Source: GADM (2024). GADM database of Global Administrative Areas, version 4.1. www.gadm.org (accessed on 1 June 2025) for (**a**); Google Earth Pro for (**b**).

The sampling sites were located in the empty area between the bottomlands of the Danjiang River and croplands (Figure 5b), where a number of typical herbaceous plant species could be found, such as *Cynodon dactylon*, *Setaria viridis*, *Artemisia argyi*, *Cirsium setosum*, *Eleusine indica*, *Artemisia annua* and *Digitaria sanguinalis*. In this study, we selected two herbaceous species from the family of Asteraceae, i.e., *Artemisia argyi* and *Cirsium setosum*, as our model species. Both species are native and commonly seen along roadsides and riverbanks in the region. They can be future candidates for revegetating species along roadsides in temperate and subtropical regions.

### 4.2. Soil and Root Sampling

Root sampling was carried out in December 2021 (within the dormant season) and September 2022 (within the late growing season). For each season, three sampling sites were randomly chosen for each species (Table 1; Figure 5b). To make sure that the sampling was representative and independent, the sites were all located within the transitional zone between croplands and the riparian zone with comparative altitude, exposition and topography. To minimize spatial autocorrelation, sampling sites for each species were spaced at a distance of at least 10 m (Figure 5b). In this study, root sampling in September and December was conducted at slightly different locations along the river (Figure 5b), which may have introduced a noisy effect of location that cannot be fully separated from the effect of season. However, we suppose that this influence on root traits is minimal, as the two locations were in close proximity and belonged to the same land use and habitat type.

At each sampling site, several mature and visually healthy individuals of the target species were carefully excavated. These individuals typically grew in clusters as dominant species within a circle zone of 1 m in diameter. The excavated soil–root composites were then transported to the laboratory, where they were fully immersed in water for 24 h prior to mechanical testing. These composites allowed us to select at least five representative individuals of the target species for mechanical testing.

### 4.3. Soil Moisture and Bulk Density Measurements

Soil moisture and bulk density were determined using the core ring method. At each sampling site, soil cylinders of 5 cm in diameter and 5 cm in depth were taken from three randomly selected undisturbed spots around the root excavation area and placed in sealed bags. The samples were then transported back to the laboratory, where they were oven-dried at 105 °C until they reached a constant weight. Soil water content was then calculated by dividing the weight loss by dry soil mass (in g g^−1^). The average water content of the three samples was recorded as the soil moisture for the site. Bulk density (in g cm^−3^) was calculated by dividing the dry soil mass by the volume of the cylinder.

### 4.4. Measurement of Root Tensile Strength

*T_r_* was measured at the individual root level. For each of the five representative plants of a species, we randomly sampled a series of intact, healthy root segments belonging to two topological orders according to the centrifugal (developmental) segment method described in Bernston [32]: first-order (i.e., closest to the stem base) and third-order lateral roots. To facilitate root sampling, the main part of an individual’s root system was spread in a tray containing clean water. Each selected root segment was carefully separated from the rest of the root system using a scalpel. The first-order and third-order lateral roots of each branch was scanned using a Brother DCP-7195DW printer. Root diameter was measured at three positions (i.e., proximal, middle and distal positions) with three replicates per position using ImageJ (1.53k) software. The procedures for root mechanical testing and trait estimation generally followed the protocol described by Mao et al. [1]. During each test, a root segment was carefully mounted on an LDW-2 (200 N) microcomputer-controlled universal testing machine (Shanghai Songdun Instrument Manufacturing Co., Ltd., Shanghai, China). The clamping orientation strictly followed the common natural growth direction of a root, with the upper clamp holding the proximal (usually thicker) end near the soil surface and the lower clamp holding the distal (usually thinner) end.

Tensile tests were conducted at a constant loading rate of 5 mm min^−1^. Prior to each test, both preload and displacement were reset to zero. The initial gauge length (distance between clamps) was precisely measured for each sample using a vernier caliper and input into the testing software. Upon test completion, the device automatically returned to its initial position, and raw data files were saved immediately. Each file was named to include key identifiers such as test number, root ID, fracture position (proximal, middle or distal) and any occurrence of slippage to facilitate data processing. To prevent slippage during testing, root diameters were secured using paper strips and adhesive. A successful test was defined as follows: (i) root failure did not occur within the clamps, and (ii) there was no slippage between the sample and the clamps. Two successful tests were performed for each root order per plant. Theoretically there were 120 tests for each season (2 species × 3 sampling sites × 5 replicate individuals × 2 root types × 2 tests). In reality, we obtained 120 tests for December and 115 tests for September due to missing samples.

Tensile strength (*T_r_*, in MPa) was calculated using the following formula:

(1) $$T_{r}=4\cdot \frac{F_{r}}{\pi d^{2}}$$

where *F_r_* is the maximum tensile force at failure (in N), and *d* is the mean root diameter (mm), which was obtained by averaging the nine diameter measurements per root.

### 4.5. Statistical Analyses

We performed t-tests to examine the effect of season on *F_r_*, *T_r_*, mean root diameter, soil moisture and bulk density. We employed power law regression [33,34] to explore the relationship between the two tensile traits (*F_r_* and *T_r_*) and diameter for each species and each season, as follows

(2) $$F_{r}=A\cdot d^{B}$$

(3) $$T_{r}=\alpha \cdot d^{\beta }$$

In these equations, *A* and *α* (coefficients) and *B* and *β* (exponents) are parameters. They were estimated using linear regressions over the logarithmic form of Equations (2) and (3) [5]. A negative exponent (*B* < 0 or *β* < 0) indicates an inverse relationship between root diameter and the mechanical trait, meaning that thicker roots exhibit lower trait values. Conversely, a positive exponent (*B* > 0 or *β* > 0) indicates a positive relationship, where thicker roots have higher mechanical trait values. If *B* or *β* is not significantly different from zero, it suggests that the mechanical trait does not vary substantially with root diameter.

To test whether the power law equations obtained from the growing season fit the dormant season data, we used the parameters of each species obtained in September to predict the root tensile traits in December and compared the predicted and observed data. To assess model precision, we plotted the predicted data against the observed data. Then, an F-test was conducted to diagnose whether the fitted linear regression line significantly deviated from the 1:1 line (no predicted bias). In addition, we plotted the residuals (i.e., the difference between observed and predicted data) against the observed data. Then, we tested whether the slope of the fitted linear regression line was significantly different from zero. All statistical analyses were conducted using R [35] (version 4.4.2; www.r-project.org, accessed on 8 June 2025) with the help of the car package [36].

## 5. Conclusions

In this study, we selected two Asteraceae species, *Artemisia argyi* and *Cirsium setosum*, which, despite belonging to the same family, exhibit striking differences in root mechanical traits. Our aim was to investigate the impact of seasonal variation on root mechanical performance. We found a high level of consistency in the seasonal responses of both species: root mechanical strength was significantly lower during the non-growing season compared to the growing season, and this effect was primarily observed in thicker, first-order roots. This may be attributed to changes in soil conditions, such as increased moisture and associated habitat alterations, which could lead to a reduction in coarse root *F_r_*.

Moreover, our results show that models calibrated using growing season *T_r_* data failed to accurately predict *T_r_* during the non-growing season. This suggests that future studies on riparian root reinforcement and slope stability should consider the necessity of incorporating dormant season *T_r_* measurements, rather than relying solely on growing season data.

As an initial exploration into seasonal variation in root mechanical traits, our study highlights the importance and potential of this research direction. Future efforts should extend seasonal measurements across a wider range of environments and plant species to determine whether seasonal variation in *T_r_* is a general phenomenon. To further enhance our mechanistic understanding of the drivers behind this variation, we advocate for integrating analyses of root microstructural changes such as anatomical features, cell organization and evidence of herbivory into future research frameworks.

## Figures and Tables

**Table 1:** Overview of sampling sites.

Month	Species	Abbreviation	Sampling Site	Latitude	Longitude	Altitude (m)
September	*Artemisia argyi*	Aa	1	33.0349° N	111.2498° E	224.6
			2	33.0353° N	111.2478° E	213.1
			3	33.0336° N	111.2523° E	215.7
	*Cirsium setosum*	Cs	1	33.0350° N	111.2498° E	223.9
			2	33.0354° N	111.2478° E	215.7
			3	33.0336° N	111.2522° E	210.5
December	*Artemisia argyi*	Aa	1	33.0688° N	111.2391° E	180.0
			2	33.0687° N	111.2390° E	182.6
			3	33.0687° N	111.2389° E	182.9
	*Cirsium setosum*	Cs	1	33.0697° N	111.2315° E	162.3
			2	33.0722° N	111.2443° E	192.6
			3	33.0707° N	111.2299° E	169.6

## Data Availability

The data presented in this paper are freely and openly accessible via the Portail Data INRAE at: https://doi.org/10.57745/WMCVFC.

## Supplementary Materials

The following supporting information can be downloaded at: https://www.mdpi.com/article/10.3390/plants14192957/s1. Figure S1: Seasonal variation in root tensile strength (*T_r_*; (a)), maximum tensile force (*F_r_*: (b)) and diameter (*d*; (c)).
