# Supplementary material for: Does Root Tensile Strength Exhibit Seasonal Variation? Evidence from Two Herbaceous Species
Source: Plants (Basel). 2025 Sep 24;14(19):2957. doi: 10.3390/plants14192957 (PMC12526503; doi:10.3390/plants14192957)

Figure S1: Seasonal variation in root tensile strength (T_r; (**a**)), maximum tensile force (F_r; (**b**)) and diameter (d; (**c**)). The terms "1st order" and "3rd order" represent the two root orders, and colors indicate September (late growing season) and December (dormant season), respectively. Significance code: *** = $p < 0.001$; ** = $p < 0.01$; * = $p < 0.05$. Outliers are included in this version.

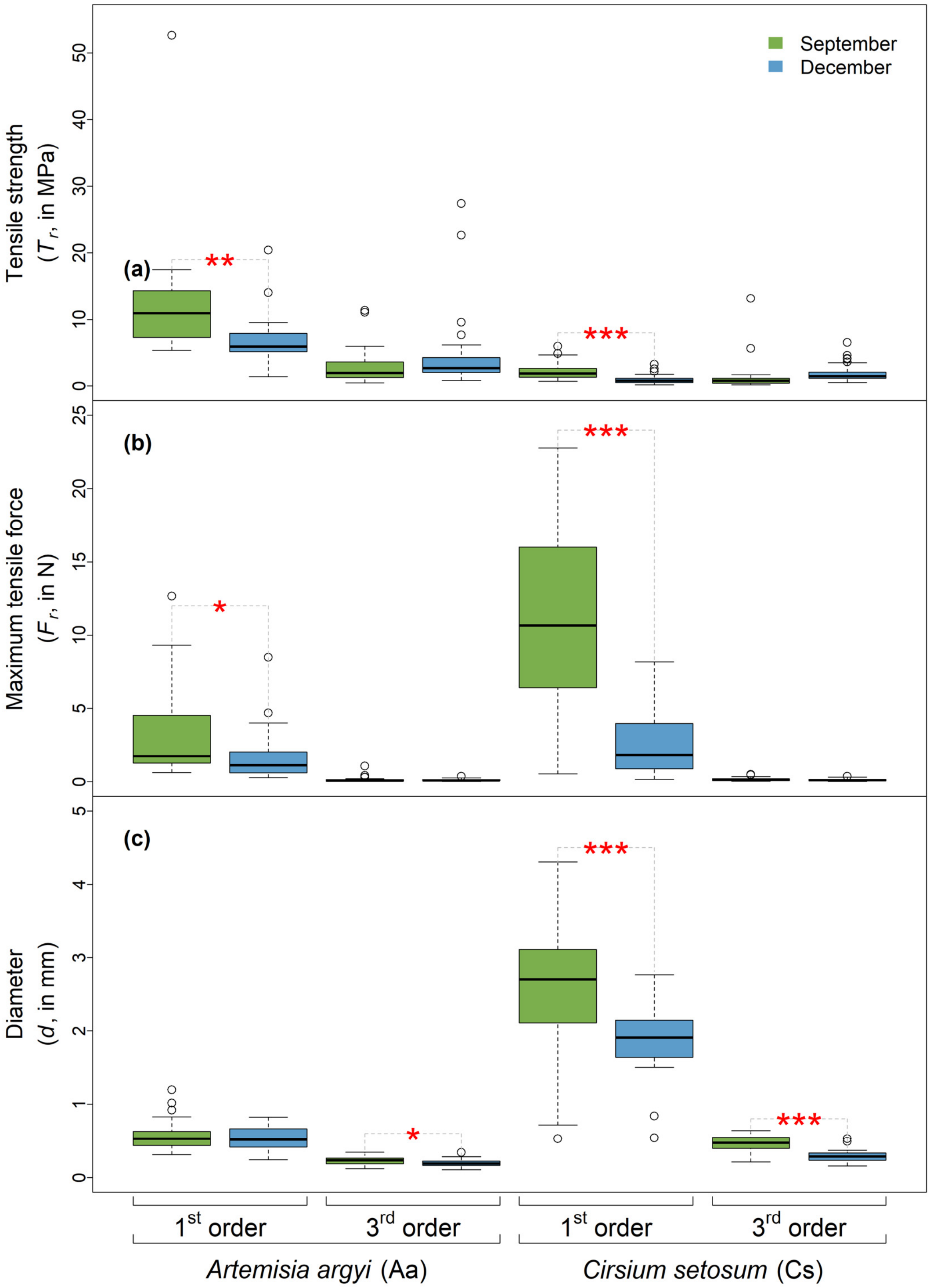

Supplement: Supplementary file 1 [file plants-14-02957-s001.zip › plants-3720475-supplementary.pdf]
